# Luteolin Induces Apoptosis and Autophagy in Mouse Macrophage ANA-1 Cells via the Bcl-2 Pathway

**DOI:** 10.1155/2018/4623919

**Published:** 2018-08-30

**Authors:** Yuexia Liao, Yang Xu, Mengyao Cao, Yuanyuan Huan, Lei Zhu, Ye Jiang, Weigan Shen, Guoqiang Zhu

**Affiliations:** ^1^Jiangsu Co-Innovation Center for Important Animal Infectious Diseases and Zoonoses, Joint International Research Laboratory of Agriculture and Agri-Product Safety of the Ministry of Education of China, Yangzhou 225009, China; ^2^College of Veterinary Medicine, Yangzhou University, Yangzhou 225009, China; ^3^College of Nursing, Yangzhou University, Yangzhou, China; ^4^College of Medicine, Yangzhou University, Yangzhou 225009, China; ^5^Jiangsu Co-innovation Center for Prevention and Control of Important Animal Infectious Diseases and Zoonoses, Yangzhou 225009, China

## Abstract

Plants rich in luteolin have been used as Chinese traditional medicines for inflammatory diseases, hypertension, and cancer. However, little is known about the effect of luteolin on the apoptosis or autophagy of the macrophages. In this study, mouse macrophage ANA-1 cells were incubated with different concentrations of luteolin. The viability of the cells was determined by an MTT assay, apoptosis was determined by flow cytometric analysis, the level of cell autophagy was observed by confocal microscopy, and the expression levels of apoptotic or autophagic and antiapoptotic or antiautophagic proteins were detected by Western blot analysis. The results showed that luteolin decreased the viability of ANA-1 cells and induced apoptosis and autophagy. Luteolin induced apoptosis accompanied by downregulation of the expression of Bcl-2 and upregulation of the expression of caspase 3 and caspase 8. And luteolin increased FITC-LC3 punctate fluorescence accompanied by the increased expression levels of LC3-I, ATG7, and ATG12, while it suppressed the expression level of Beclin-1. Luteolin treatment resulted in obvious activation of the p38, JNK, and Akt signaling pathways, which is important in modulating apoptosis and autophagy. Thus, we concluded that luteolin induced the apoptosis and autophagy of ANA-1 cells most likely by regulating the p38, JNK, and Akt pathways, inhibiting the activity of Bcl-2 and Beclin-1 and upregulating caspase 3 and caspase 8 expression. These results provide novel insights into a therapeutic strategy to prevent and possibly treat macrophage-related diseases through luteolin-induced apoptosis and autophagy.

## 1. Introduction

Macrophages fulfill a broad range of functions in phagocytosis, microbial killing, host defense, tissue homeostasis and repair, pathology and development, and their proliferation; migration and apoptosis have been emerged as important therapeutic targets for a variety of human diseases [1, 2]. Macrophages are critically involved in diseases that are caused by chronic inflammation (e.g., arthritis, multiple sclerosis, inflammatory bowel diseases, atherosclerosis, and cardiovascular disease) [3–6]. In addition, the apoptosis and autophagy dysfunction of the macrophage impact the development of chronic inflammation (e.g., atherosclerosis, tuberculosis, and sepsis) [7–10].

Plants rich in luteolin (3′,4′,5,7-tetrahydroxylavone), an active polyphenolic compound, have been used as Chinese traditional medicine to treat inflammatory diseases, hypertension, and cancer for a long period of time [11]. The pharmacological activities of luteolin, such as antioxidant, radical scavenging, cytoprotective, anti-inflammatory, antiallergic, and antitumor properties [12, 13], have been observed, suggesting that luteolin might possess diverse health benefits for treating some diseases, such as allergies, cancer, respiratory diseases, and cardiovascular health [14]. Moreover, it has been reported that luteolin can induce apoptosis in a wide variety of cancer cells *in vitro*, including prostate cancer cells, esophageal carcinoma cells, gastric cancer cells, and colon cancer cells [15–17]. Despite the well-documented antioxidant, anti-inflammatory, and antineoplastic potential of luteolin, little is known about the effect of luteolin on the apoptosis or autophagy of the macrophages.

The results of this study revealed that luteolin induced apoptosis and autophagy of the mouse macrophage ANA-1 cells via altering the cell viability and DNA fragment. Moreover, luteolin improved apoptosis and autophagy of the macrophage presumably through the Bcl-2- and MAPK-mediated signaling pathways. The results suggested that the understanding of the molecular mechanisms underlying luteolin-induced apoptosis and autophagy may be useful in cancer therapeutics, chemoprevention, and treating inflammation and neurodegenerative diseases, such as Parkinson's disease and Alzheimer's disease [18].

## 2. Materials and Methods

### 2.1. Reagents and Antibodies

Luteolin (HPLC > 95%) was purchased from Nanjing TCM Institute of Chinese Materia Medica and dissolved in sodium carbonate (1 mM) stock solutions and stored at −20°C. Purified rabbit anti-Akt, anti-phospho-Akt, anti-phospho-ERK1/2, anti-ERK1/2, anti-phospho-p38, anti-p38, anti-*β*-actin, and HRP-linked anti-rabbit IgG were purchased from Cell Signaling Technology (USA). Apoptosis/Necroptosis Antibody Sampler Kit and Autophagy Antibody Sampler Kit were purchased from Cell Signaling Technology (USA). Annexin V-FITC Apoptosis Detection Kit was purchased from Beyotime Institute of Biotechnology (Shanghai, China). In Situ Cell Death Kit, Fluorescein, was purchased from Roche Applied Science (Mannheim, Germany).

### 2.2. Cell Culture

ANA-1 macrophages, obtained from the Cell Bank of Shanghai Institute of Biochemistry and Cell Biology of the Chinese Academy of Sciences (Shanghai, China), were maintained in RPMI 1640 medium supplemented with 10% fetal bovine serum (FBS), 100 units/ml penicillin, and 100 *μ*g/ml streptomycin and cultured at 37°C in a humidified atmosphere of 6% CO_2_.

### 2.3. Cell Viability Assay

Cell viability was assessed by using the MTT (3-(4,5-dimethylthiazol-2-yl)-2,5-diphenylterazolium bromide) assay as described previously [19]. In brief, 200 *μ*l of 2 × 10^5^ cells in RPMI 1640 medium with 0, 5, 10, 20, 40, 80, or 160 *μ*M luteolin was seeded in 96-well culture plates and cultured at 37°C in 6% CO_2_ for 24 h or 48 h and was incubated with 50 *μ*l of MTT solution (5 mg/ml in PBS) for another 4 h at 37°C. Subsequently, the MTT solution was removed from the wells by aspiration and the formazan crystals were dissolved in 100 *μ*l of DMSO. The absorbance at 570 nm was measured with a Bio-Tek MQX 680 (Bio-Tek Instruments Inc., Winooski, VT, USA). The data are described as mean ± standard deviations (SD) from at least five independent experiments.

### 2.4. Analysis of Cell Apoptosis

For assays measuring the induction of apoptosis of the ANA-1 cells by luteolin, the ANA-1 cells were incubated with 0, 5, 10, 20, and 40 *μ*M of luteolin at 37°C in 6% CO_2_ for 24 h or 48 h and then assayed for apoptosis using the techniques listed below as described previously [19]. For the quantification of the cells in the sub-G1 stages based on DNA content, cells were fixed in ethanol (70%) at 4°C overnight and stained with PI (50 *μ*g/ml) and RNAse (10 *μ*g/ml) at 37°C in the dark for 30 min and subsequently quantified with flow cytometry (BD Biosciences, San Jose, CA, USA). For detecting cell surface phosphatidylserine, cells were dual stained with FITC-conjugated Annexin V and PI using an Annexin V-FITC Apoptosis Detection Kit according to the manufacturer's instructions. Apoptosis was further confirmed by the TUNEL assay, which detects the DNA fragmentation that is the characteristic of apoptosis, using an In Situ Cell Death Kit, Fluorescein (Roche Applied Science, Mannheim, Germany), following the manufacturer's guideline. The TUNEL-positive cells were evaluated by flow cytometry. In addition, the levels of caspase 3, Bax, and Bcl-2 were determined by Western blot analysis as described below.

### 2.5. Cell Autophagy Assay

To analyze autophagic flux, the ANA-1 cells were seeded on glass cover slips in 24-well plates with 0, 5, 10, 20, or 40 *μ*M luteolin for 48 h. Routinely, cells were washed with PBS, fixed with ice cold 4% paraformaldehyde for 30 min at 4°C and blocked with 5% BSA in PBS for 1 h. Cells were incubated with primary antibody to LC3 (1 : 500 dilution anti-Rabit-LC3-pAb, MBL, Japan) overnight at 4°C and then incubated with FITC-goat-anti-rabbit-IgG (ZSJB-BIO, Beijing, China) for 30 min at 37°C in the dark; DAPI (Beyotime Institute of Biotechnology, Shanghai, China) counterstain was used for nuclear staining. After extensive washing, the cover slips were then mounted on glass slides and the fluorescent images were captured with confocal microscopy (TCS SP8 STED, Leica, German). Also, the averaged intracellular autophagosome was counted as the intracellular gathering FITC-LC3 spots divided the cell number dyed with DAPI in 5 random fields at a magnification of ×1000 in different cover slips. The levels of LC3, Beclin-1, ATG7, and ATG12 were determined by Western blot analysis as described below.

### 2.6. Western Blot Analysis

The cells were treated as mentioned above and lysed in cell lysis buffer (Beyotime Institute of Biotechnology, Shanghai, China) containing 1 *μ*M phenylmethylsulfonyl fluoride, 1.5 *μ*M pepstatin A, and 0.2 *μ*M leupeptin. The samples were separated by 10% SDS-polyacrylamide gel electrophoresis and were then transferred to polyvinylidene fluoride membranes, which were blocked with 5% dried milk protein using the techniques listed below as described previously [19]. After blocking, the blots were incubated with primary antibodies. After being washed with TBST, the membranes were incubated at 37°C for 1 h in blocking buffer that contained the secondary antibodies and were visualized via a chemiluminescence ECL Western blotting analysis system (BD, USA). The protein levels were quantified using ImageJ software (NIH, USA) and are expressed as percentages of the control after normalization to the housekeeping protein *β*-actin.

### 2.7. Statistical Analyses

The quantitative data are expressed as mean ± SDs or SEM. The data were analyzed using SPSS 19.0 software. Statistical analyses were performed with one-way analyses of variance (ANOVA) followed by Tukey's multiple comparison post hoc tests. *p* values below 0.05 were considered statistically significant.

## 3. Results

### 3.1. Luteolin Decreases ANA-1 Cell Viability

**Figure 1:**
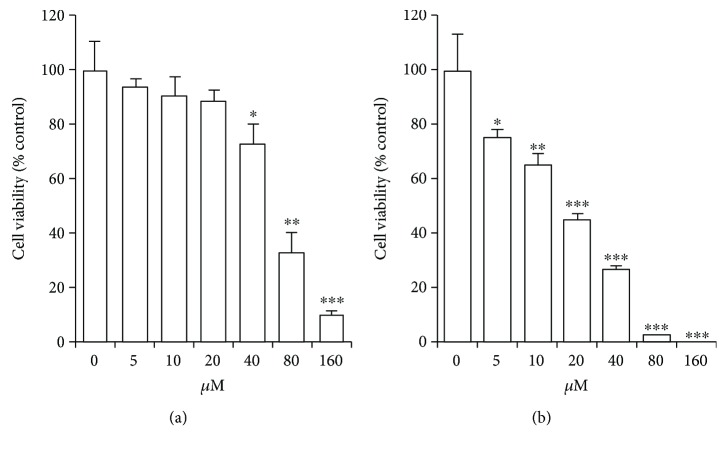
The cell viability of ANA-1 cells treated with luteolin. (a) Cell viability was analyzed with MTT assay after exposing to luteolin for 24 h. (b) Cell viability was analyzed with MTT assay after exposing to luteolin for 48 h. All data are expressed as the percentage of change in comparison with that of the control group, which were assigned 100% viability. The values are given as mean *±* SD (*n* = 5). ^∗^*p* < 0.05, ^∗∗^*p* < 0.01, and ^∗∗∗^*p* < 0.01 versus the control.

To examine the effects of luteolin on cell viability, the ANA-1 cells were exposed to different concentrations (0, 5, 10, 20, 40, 80, and 160 *μ*M) of luteolin for 24 h or 48 h and cell viability was measured by MTT assay. As shown in Figure 1, luteolin significantly suppressed the viability of ANA-1 cells in a dose- and time-dependent manner. After 48 h of incubation with 20 *μ*M luteolin, the viability of the cells was reduced by 55% approximately. Moreover, 80 *μ*M luteolin reduced cell viability by 73% or more after 24 h or 48 h of treatment. These results indicate that luteolin significantly decreases the viability of ANA-1 cells, and the half maximal inhibitory concentration (IC50) was about 40 *μ*M in the cell line. Thus, we chose 5 *μ*M to 40 *μ*M as experimental concentrations in further experiments to avoid severe cytotoxic side effect.

### 3.2. Luteolin Induces ANA-1 Cell Apoptosis via Modulating the Expression of Caspase 3 and Bcl-2

**Figure 2:**
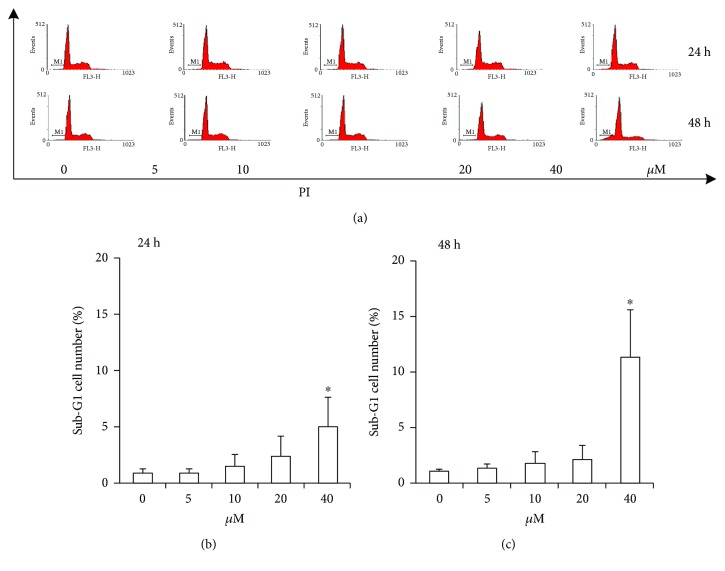
Luteolin induces the change of DNA content in the ANA-1 cells. (a) The representative image from flow cytometry to analyze cellular DNA content. (b) The statistical analysis of the data from (a) to show the cellular DNA content at 24 hours post treatment. (c) The statistical analysis of the data from (a) to show the cellular DNA content at 48 hours post treatment. The values are shown as mean ± SEM. ^∗^*p* < 0.05 versus the control. ANA-1 macrophages were exposed to different concentrations of luteolin for 24 h or 48 h. The DNA content of ANA-1 cells in sub-G1 was assayed by flow cytometry.

**Figure 3:**
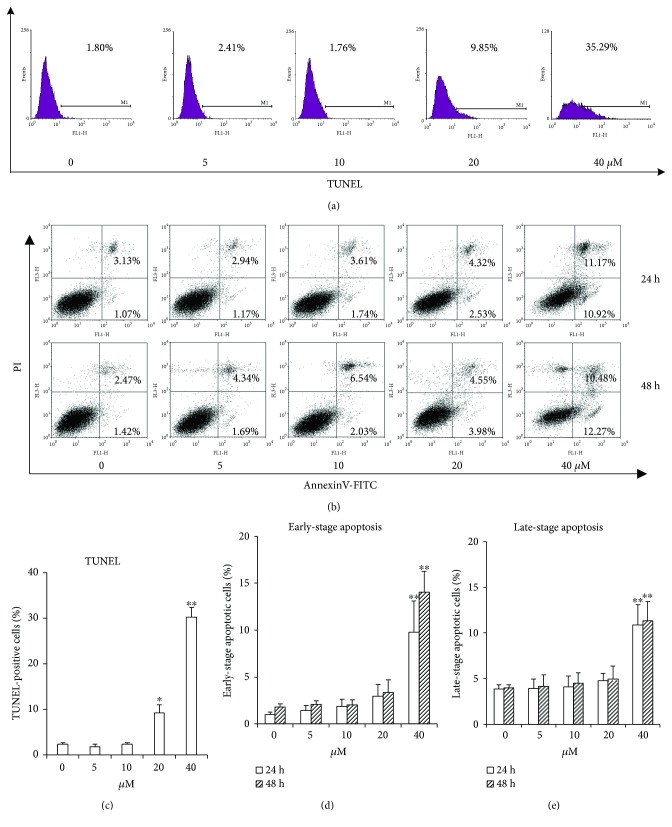
Luteolin induces the ANA-1 macrophages apoptosis using TUNEL assay and Annexin V-FITC/PI staining. (a) The images of TUNEL-positive cells were captured by FCM. (b) The flow cytometry data show one representative Annexin V-FITC/PI staining result. (c) The ANA-1 macrophages were exposed to different concentrations of luteolin for 48 h. The values are given as mean ± SD.^∗^*p* < 0.05 and ^∗∗^*p* < 0.01 versus the control. (b) The flow cytometry data show one representative TUNEL assay result. (d-e) The ANA-1 macrophages were exposed to different concentrations of luteolin for 24 h and 48 h. The rates of early- and late-stage apoptotic cell death were detected by staining cells with Annexin V-FITC and PI and analyzing by flow cytometry. The values are given as mean ± SD. ^∗^*p* < 0.05 and ^∗∗^*p* < 0.01 versus the control.

**Figure 4:**
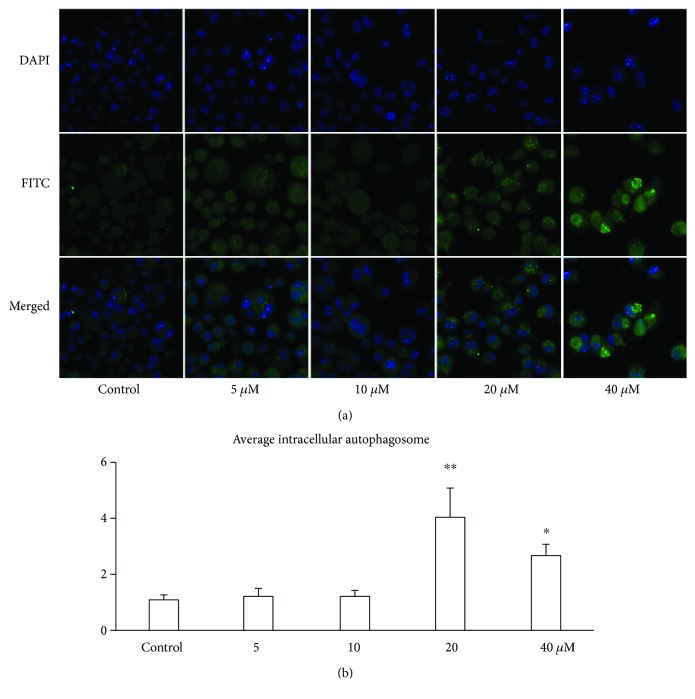
Luteolin increased the ANA-1 macrophage autophagosome using immune fluorescence combined with CLSM. (a) Immunofluorescence staining of LC3.The ANA-1 cells were seeded on glass cover slips in 24-well plates with luteolin for 48 h. The fluorescence images were captured with a confocal microscopy. Magnification, ×1000. (b) Average intracellular autophagosome in macrophages treated with luteolin for 48 h. The values are given as mean ± SEM. ^∗^*p* < 0.05 and ^∗∗^*p* < 0.01 versus the control.

To verify whether the observed inhibition of cell viability induced by luteolin is the consequence of the induction of apoptosis, we evaluated the effect of luteolin on ANA-1 cell apoptosis. First, we investigated the effect of luteolin on DNA fragmentation in ANA-1 cells after incubation for 24 h or 48 h. The rates of macrophages at sub-G1 (Figure 2) and the TUNEL assay (Figure 3(a)) revealed that luteolin significantly increased DNA fragmentation in the ANA-1 cells. DNA fragmentation in medium containing 40 *μ*M luteolin showed a significantly higher percentage than that in the control group. Specifically, 40 *μ*M luteolin induced the rate of ANA-1 cells at sub-G1 by 15% after 48 h incubation (Figure 2). Second, we detected the effect of luteolin on cell apoptosis induction of ANA-1 cells by measuring the rates of macrophages at early- and late-stage apoptotic cells. As shown in Figures 3(b) and 3(c), the same luteolin concentration gradient induced ANA-1 cell early and late stages of apoptosis in a dose-dependent manner after treatment for 24 h or 48 h. Finally, the data from Western blot analysis showed that, after incubation with luteolin for 48 h, luteolin (40 *μ*M) significantly increased the levels of caspase 3 and downregulated the expression of the antiapoptotic molecule Bcl-2 but did not significantly affect the level of Bax (Figures 4(a) and 4(b)). These results demonstrated that luteolin decreased the viability of ANA-1 cells partially through the induction of apoptosis by modulating the expression of caspase 3 and Bcl-2.

### 3.3. Luteolin Induces ANA-1 Cell Autophagy

**Figure 5:**
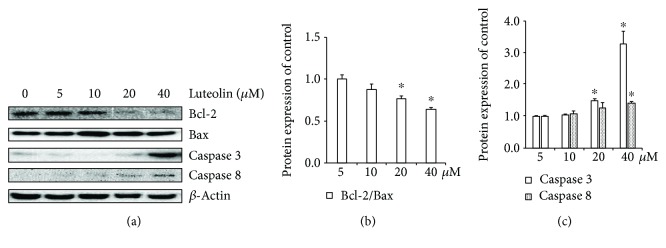
The effects of luteolin on apoptotic and antiapoptotic proteins in ANA-1 cells. (a) Representative Western blot results showed the rate of Bcl-2/Bax, caspase 3, and caspase 8 expression in the ANA-1 cells after incubation with luteolin for 48 h (b and c). The relative expression of proteins compared with the control. The values are given as mean ± SEM.^∗^*p* < 0.05 versus the control.

**Figure 6:**
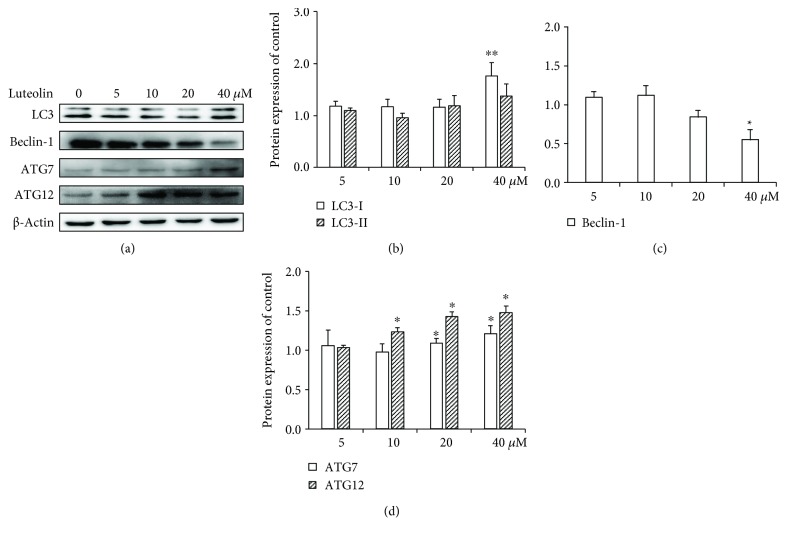
The effects of luteolin on autophagic and antiautophagic proteins in ANA-1 cells. (a) Representative Western blot results showed LC3, Beclin-1, ATG7, and ATG12 expression in the ANA-1 cells after incubation with luteolin for 48 h. (b–d) The relative expression of proteins compared with the control. The values are given as mean ± SEM.^∗^*p* < 0.05 and ^∗∗^*p* < 0.01.

Since we observed luteolin-induced apoptosis in ANA-1 cells via modulating the expression of caspase 3 and Bcl-2, which is also essential for cell autophagic activity [20], we next examined the effect of luteolin on the autophagy of ANA-1 cells using confocal microscopy and Western blot analysis. As show in Figure 5, luteolin at the same concentration gradient significantly increased intracellular gathering FITC-LC3 spots. The levels of LC3-I, Beclin-1, ATG7, and ATG12 were detected by Western blot analysis after incubation with luteolin for 48 h. As shown in Figure 6, after ANA-1 cell incubation with 40 *μ*M of luteolin for 48 h, the levels of Beclin-1 were significantly decreased by 45% and those of LC3-I, ATG7, and ATG12 were significantly increased by 76%, 21%, and 48%, respectively, when compared with those of the control group. In parallel, luteolin has no significant effect on the expression of LC3-II. These results suggested that luteolin induced macrophage autophagy by regulating the balance between Bcl-2 and Beclin-1 and then upregulating LC3, ATG7, and ATG12 levels.

### 3.4. ERK, P38, and Akt Phosphorylation during ANA-1 Apoptosis and Autophagy

**Figure 7:**
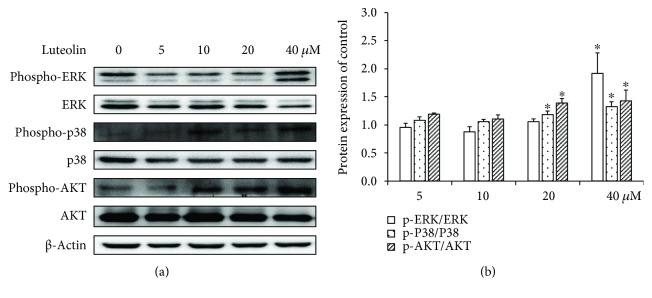
The effects of luteolin on ERK/p38/Akt in the ANA-1 cells. (a) Representative Western blot results showing phospho-ERK1/2/ERK1/2, phospho-p38/p38, and phospho-Akt/Akt expression in the ANA-1 cells. (b) The relative expression of proteins compared with the control. ^∗^*p* < 0.05 versus the control.

Next, we investigated the mechanisms for the apoptosis and autophagy-inducing effect of luteolin in ANA-1 cells. We examined the phosphorylation levels of Akt, ERK, and p38 MAPK that are upstream signaling molecules of regulating cell apoptosis and autophagy pathways and play a vital role in cellular apoptosis and autophagy. As shown in Figure 7, luteolin treatment increased the levels of phospho-ERK, phospho-P38, and phospho-Akt in ANA-1 cells in a concentration-dependent manner compared to those of the control cells. Exposure of ANA-1 cells to 40 *μ*M luteolin for 48 h increased the phospho-ERK, phospho-P38, and phospho-Akt levels by 127%, 32%, and 51%, respectively. However, incubation of ANA-1 cells with luteolin did not significantly affect the expression of total ERK, P38, and Akt. The results suggested that luteolin induced the apoptosis and autophagy of ANA-1 cells via the Akt and MAPK signaling pathways.

## 4. Discussion

Several studies have reported that luteolin induced apoptosis in a wide variety of cancer cells *in vitro*, regulated the cell cycle, exerted antiangiogenesis activity, and exhibited anti-inflammatory effects [12, 14–17]. Here, we tried to discover the role of luteolin in the apoptosis and autophagy in macrophage. The results of this report revealed that luteolin significantly increased the ANA-1 macrophage apoptosis and autophagy via inhibiting the expression of Bcl-2 and Beclin-1 and increasing the expression of caspase 3 and caspase 8.

In the current study, the results from MTT assay showed that luteolin could decrease the viability of the ANA-1 cell line in a dose- and time-dependent manner *in vitro*. Also, the results from flow cytometry demonstrated that 40 *μ*M luteolin could induce the early-stage and late-stage apoptois in the ANA-1 cells. These are consistent with the findings from other groups in cancer cells. In their studies, they reported that luteolin could induce growth inhibition and apoptosis in a dose- and time-dependent manner with estimated IC50 ranging from around 35 to around 70 *μ*M [15, 16, 18]. The outcomes of macrophage apoptosis depend on the balance of activities between apoptotic and antiapoptotic factors [21]. Bcl-2, Bax, caspase 3, and caspase 8 are important proteins that participate in cell apoptosis. Bcl-2 antiapoptotic protein inactivation increases the susceptibility of cells to apoptosis while caspase 3 and caspase 8 play a central role in the execution phase of cell apoptosis. Both the extrinsic and intrinsic pathways converge at caspase 3 as one of the last steps in cell death [22, 23]. These data suggested that luteolin induced apoptosis via upregulating caspase 3 and caspase 8 by downregulating the rate of Bcl-2/Bax.

It is well known that Bcl-2 can affect the balance between cellular apoptosis and autophagy by binding or dissociating its antiapoptotic and antiautophagy partner beclin-1, a central role in incipient autophagy [22, 24]. Autophagy is an intracellular degradation system that delivers cytoplasmic constituents into the lysosome [25]. It has been demonstrated that apoptosis and autophagy often occur in the same cell and can interact with each other [22, 26]. The activation of caspase-3 also can lead to the cleavage of autophagy protein Beclin-1, which results in the inactivation of the autophagic program and cell apoptosis [22]. In this study, we found that luteolin increased LC3 assembly and the expression of LC3-I and decreased expression of Bcl-2 and Beclin-1 in the ANA-1 cells, suggesting that luteolin induced the ANA-1 cell apoptosis via downregulating Bcl-2 and Beclin-1 and upregulating caspase 3 and caspase 8, subsequently causing macrophage autophagy.

The activations of Akt, p38 and ERK are involved in cell apoptosis and autophagy and play a key role in the crosstalk between autophagy and apoptosis [27–29]. The activation of p38 augments apoptosis and mediates autophagy in response to chemotherapeutic agents [29, 30].

ERK signaling is classically known as an important regulator of cell survival [31]. ERK activity can mediate cell apoptosis and autophagy *in vitro* and *in vivo* [32]. Luteolin can regulate the expression of phospho-ERK in different cells [33–35]. The PI3K/Akt signaling pathway plays a critical regulatory role in many cellular processes including RNA processing, translation, autophagy, and apoptosis [36, 37].

In this study, the upregulation of phospho-p38, phospho-ERK, and phospho-Akt was shown in the ANA-1 cells after treatment with luteolin, suggesting that luteolin induces ANA-1 cell apoptosis and autophagy presumably via the activation of the p38, ERK, and Akt pathways.

In summary, luteolin effectively improved apoptosis and autophagy of the ANA-1 macrophage most likely by regulating the p38, ERK, and Akt pathways to inhibit the expression of Bcl-2 and Beclin-1 and increase the expression of caspase 3 and caspase 8. The results provide novel insights into a therapeutic strategy to prevent and possibly treat macrophage-related diseases through luteolin-induced apoptosis and autophagy.

## Data Availability

The data used to support the findings of this study are available from the corresponding author upon request.
